# Sex differences in sensation-seeking: a meta-analysis

**DOI:** 10.1038/srep02486

**Published:** 2013-08-30

**Authors:** Catharine P. Cross, De-Laine M. Cyrenne, Gillian R. Brown

**Affiliations:** 1 University of St Andrews, Fife, UK; 2 California State University, Sacramento, USA

## Abstract

Men score higher than women on measures of sensation-seeking, defined as a willingness to engage in novel or intense activities. This sex difference has been explained in terms of evolved psychological mechanisms or culturally transmitted social norms. We investigated whether sex differences in sensation-seeking have changed over recent years by conducting a meta-analysis of studies using Zuckerman's Sensation Seeking Scale, version V (SSS-V). We found that sex differences in total SSS-V scores have remained stable across years, as have sex differences in Disinhibition and Boredom Susceptibility. In contrast, the sex difference in Thrill and Adventure Seeking has declined, possibly due to changes in social norms or out-dated questions on this sub-scale. Our results support the view that men and women differ in their propensity to report sensation-seeking characteristics, while behavioural manifestations of sensation-seeking vary over time. Sex differences in sensation-seeking could reflect genetically influenced predispositions interacting with socially transmitted information.

Sensation-seeking is a personality trait reflecting the desire to pursue novel or intense experiences, even if risks are involved^1^. Questionnaire measures of sensation-seeking ask people whether they would like to try adventurous activities, such as extreme sports or travelling to remote places; whether they enjoy loud parties and speaking in front of groups; and whether they dislike dull or repetitive activities, such as standing in queues^2^,^3^. People who score high on self-report measures of sensation-seeking have a higher propensity to misuse drugs, engage in risky sexual activities, and suffer accidental injuries than low sensation-seekers^4^. Understanding individual variation in sensation-seeking is therefore useful in creating targeted interventions to improve health and wellbeing^5^.

Men tend to have higher average scores than women on questionnaire measures of sensation-seeking, such as Zuckerman's widely used Sensation Seeking Scale, version V (SSS-V)^1^,^3^, and this sex differences has been reported across populations. For example, studies conducted in the USA, Europe, Australia and China have all reported higher average scores in men than women on three of the four sensation-seeking subscales, namely Thrill and Adventure Seeking (TAS; interest in physically challenging activities), Disinhibition (Dis; favourable attitudes to uninhibited social interactions), and Boredom Susceptibility (BS; dislike for repetition and predictability), while not differing on Experience Seeking (ES; interest in low-risk, novel experiences)^6^,^7^. Men also have higher average scores than women on related measures of risk-taking, defined as behaviour that could lead to undesirable or damaging outcomes^8^.

Evolutionary psychologists have argued that men and women differ in both sensation-seeking and risk-taking as a result of sex differences in selection pressures acting on our human ancestors^9^,^10^. Selection is argued to favour risky, adventurous strategies in males when the variance in male reproductive success exceeds that of female reproductive success, if such strategies provide males with an advantage in competition for resources or mating opportunities^9^,^11^. In contrast, higher levels of parental investment in offspring have been argued to favour more risk-averse strategies in females than in males^12^. Such evolutionary hypotheses are consistent with evidence that physiological sex differences are linked to sensation-seeking; for example, testosterone levels have been reported to correlate positively with sensation-seeking scores in some studies^13^, although not in others^14^.

Other researchers have focused on how sex differences in personality, behaviour and preferences are shaped by gender roles and sexual stereotypes, which can change over time^15^. In support of the importance of social factors, a meta-analysis of studies on risk-taking has shown that sex differences have declined over recent decades^8^. Sex differences in other behavioural and cognitive traits have also declined over time, including sex differences in verbal abilities^16^ and attitudes towards casual sex^17^. Such year-of-publication effects have been attributed to changing gender stereotypes and an increase in traditionally ‘masculine' traits being reported by women^15^,^18^. However, interpretation of these year-of-publication effects is often made difficult by the lack of a common metric across studies, which means that the year of publication is confounded with changes in measurement instrument^19^.

Here, we examine whether sex differences in sensation-seeking vary according to publication date using meta-analytic techniques. We focus on Zuckerman's SSS-V scale^1^,^3^. This measure has been available since the late 1970s and remains in frequent use, even though more recent measures have been developed (e.g. Arnett Inventory of Sensation Seeking^2^; Brief Sensation Seeking Scale^20^; Impulsive Sensation Seeking Scale^21^). The SSS-V thus provides an opportunity to examine effect sizes across a 35-year period for a single measurement instrument. From an evolutionary psychology perspective, sex differences in sensation-seeking would be predicted to remain relatively stable over time, based on the argument that sex differences in self-reported personality traits reflect evolved psychological mechanisms^22^,^23^, or, alternatively, to increase over time, if the relaxation of sexual stereotypes allows underlying predispositions to be expressed more strongly^24^. In contrast, a socialisation perspective^15^ would predict that sex differences in sensation-seeking are most likely to have declined across time, if the flexibility of gender roles has increased in the study populations during this time period. Most studies using the SSS-V have been carried out in English-speaking, Westernised cultures, in which gender roles are likely to have become less constrained since the 1970s. We examined whether sex differences in sensation-seeking scores have decreased, increased or remained stable during this time period.

## Results

Our literature search (see Methods) retrieved 72 articles with appropriate data on sensation-seeking published between 1978 and 2012, and the total number of effect sizes was 323.

### Weighted effect sizes

**Figure 1:**
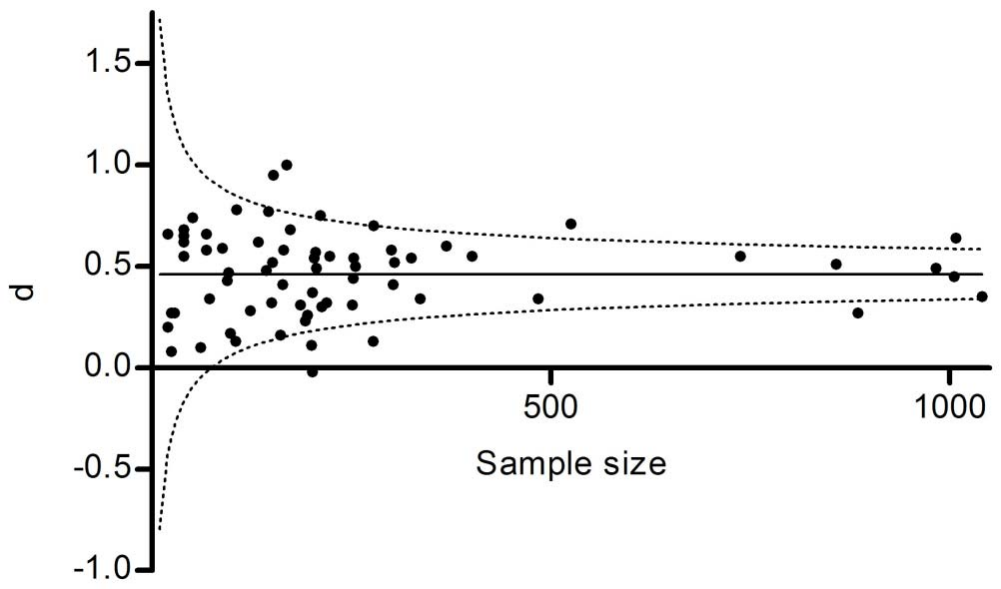
Effect sizes (d) by sample size for Total SSS-V scores. Solid line = weighted mean effect size; dashed lines = 95% confidence intervals for mean effect size.

Men scored higher than women on total SSS-V (d = 0.46; Figure 1). Effect size did not correlate with the study's inverse variance (tau-b = −0.041, n.s.), and the funnel plot was symmetric, indicating that our dataset did not suffer from publication bias (see Figure S2 in the Supplementary Information for funnel plots and tests for each subscale). Men also scored higher than women on TAS (d = 0.42), and Dis (d = 0.46), which both showed moderate sex differences. Men scored higher on BS, although this difference was smaller (d = 0.35). ES showed no sex difference (d = 0.04; Table 1).

### Changes in effect size over time

**Figure 2:**
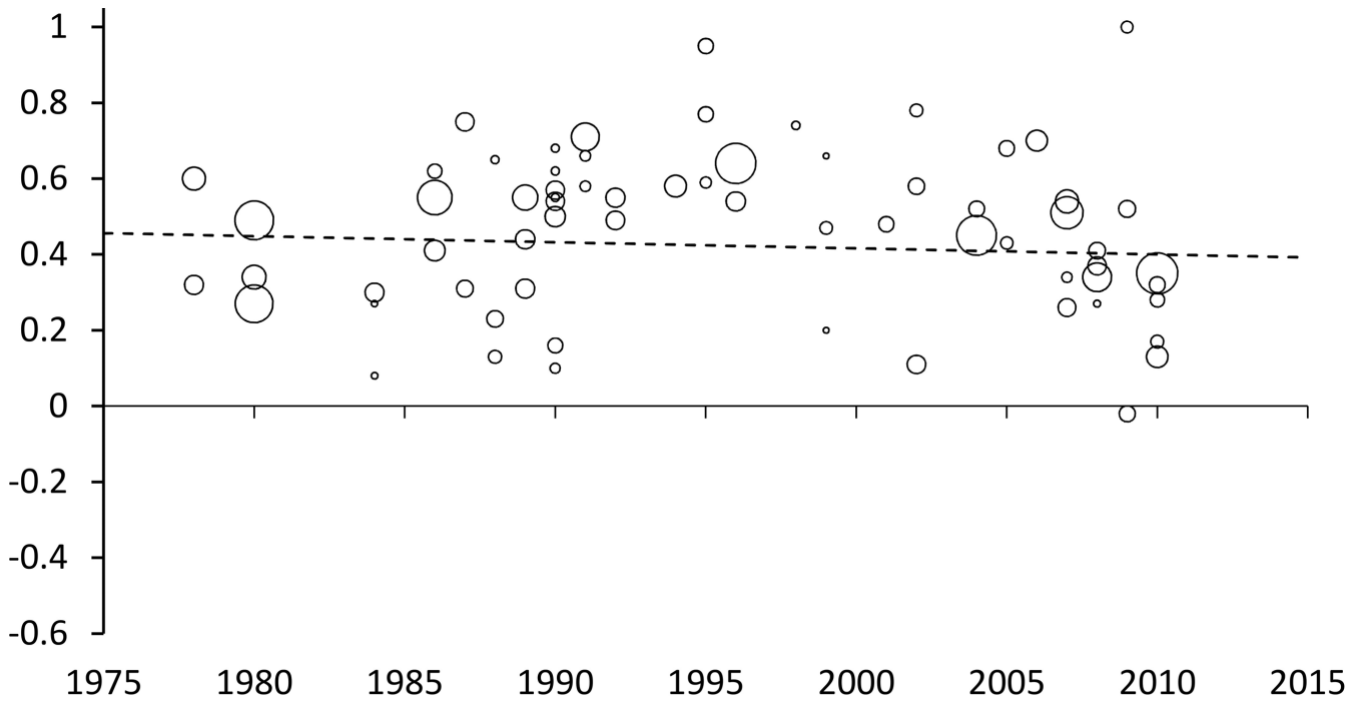
Sex differences in sensation-seeking (Cohen's d) by study year for total SSS-V scores. Bubbles are scaled to inverse study variance.

**Figure 3:**
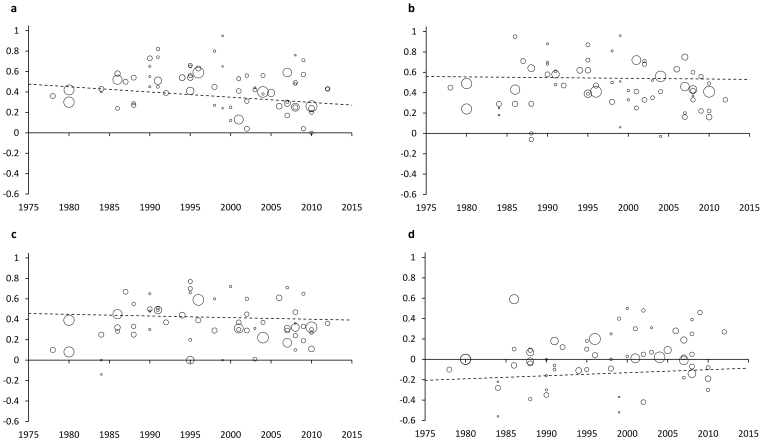
Sex differences in sensation-seeking (Cohen's d) by study year for: a) TAS, b) Dis, c) BS, and d) ES. Bubbles are scaled to inverse study variance.

Sex differences in total SSS-V scores have not diminished across time (β = −0.002, SE = 0.002, n.s.; Figure 2). In contrast, the sex difference in TAS has become significantly smaller (β = −0.005, SE = 0.002, p = .02, Beta = −0.28, R^2^ = 0.08; Figure 3a). The size of the sex difference has reduced by more than a third in the last 35 years (our maximum likelihood model gives d values of 0.48 for 1978 and 0.28 for 2013). The diminishing sex difference in TAS results from a reduction in men's scores across time: the slope of the regression line was significant and negative for male scores (B = −0.034, SE = 0.009, p < .001, Beta = −0.43, R^2^ = 0.18), while the slope for women's scores did not differ from zero (B = −0.017, SE = 0.011, n.s.). None of the other subscales showed evidence of a change in effect size with study year (Dis: β = −0.001, SE = 0.003, n.s.; BS: β = −0.001, SE = 0.003, n.s.; ES: β = −0.003, SE = 0.003, n.s.; Figure 3b–d) (Section 3 of the Supplementary Information and Figure S1 show scores over time for all subscales).

## Discussion

The results of our analyses show that sex differences on Zuckerman's Sensation Seeking Scale, version V, have generally not declined over the past 35 years. The mean effect size for the sex difference in total SSS-V scores was moderate and stable across the years of study (1978–2012). Sex differences in BS and Dis were small to medium and were also stable across time, while a sex difference in ES, which assesses openness to experiences that do not entail physical or social risk, has been consistently absent. However, the sex difference in TAS scores has diminished significantly over the years of study, due to a decrease in men's scores. Overall, these findings support the view that males and females differ in their average propensity to report sensation-seeking characteristics, while interest in specific sensation-seeking activities may vary according to changing circumstances.

Our finding that the sex difference in TAS scores has become smaller over recent years supports the argument that cultural factors can affect self-reported traits. Previous meta-analyses have reported that sex differences in other personality traits have also diminished across time^18^,^25^. The reduction in the sex difference in TAS scores could result from changes in socialisation patterns that have led to physically adventurous activities becoming less ‘male-typed', with male and female scores thus becoming more similar in recent years. This interpretation is consistent with evidence that participation in college sports is becoming more gender balanced across time in response to concerted efforts to encourage female sports participation^26^. However, our analyses show that the pattern of results is not due to an increase in female TAS scores across time, but rather a decline in male scores. Women could be showing a greater willingness to engage in thrill and adventure seeking relative to men over time, while changes in absolute scores are being influenced by other factors, such as average fitness levels.

Alternatively, the reduction in the sex difference could result from specific questions on the TAS subscale becoming out-dated; for example, several of the challenging physical activities used in the questionnaire (e.g., skiing) may no longer be viewed as novel or intense^2^, which could result in male and female scores converging towards a floor effect. Our pattern of results is consistent with participants reporting lower interest in engaging in activities that are no longer considered exciting or that participants have already tried. From our analyses, however, we cannot determine whether the declining sex difference in TAS scores is more likely to result from out-dated questions or from changes in socialisation patterns. In addition, out-dated items could arguably have led to both male and female scores increasing over time, if participants are more willing to report interest in engaging in activities that are familiar. Future research could examine the effects of redesigning the TAS sub-scale with up-to-date extreme sports activities, or researchers could use alternative measures (e.g., Impulsive Sensation Seeking Scale^21^). Revised sensation-seeking scales could also ask participants whether they are interested in experiencing some of the physical measures associated with risky activities, such as high heart rate and breathlessness, rather than refer to specific activities.

The stable sex differences in self-reported Dis and BS are consistent with the hypothesis that males exhibit stronger predispositions to engage in uninhibited social interactions and avoid repetitive activities than females, and that such differences are not attributable solely to male and female gender roles. Previous meta-analyses have also reported evidence for sex differences in related personality traits; for example, on average, men score higher than women on measures of venturesomeness^27^, while women score higher than men on harm-avoidance^28^. A comprehensive meta-analysis of the impulsivity literature concluded that the evidence for sex differences in personality traits related to sensation-seeking is strongest for low-level, emotional processes, such as sensitivity to punishment, rather than specific, higher-level cognitive processes^27^. We therefore suggest that sex differences in personality traits related to sensation-seeking and risk-taking might have domain-general emotional processes as their substrate.

Stable sex differences in Dis and BS could also be compatible with a socialisation perspective if the socially transmitted social norms and stereotypes that promote sensation-seeking in males rather than females have remained stable across years. While critics have argued that this kind of scenario is unlikely^24^, ‘social role theory' provides a framework by which some social norms are more likely than others to emerge and to remain stable over time^29^. This theory proposes that physical differences between the sexes, such as gestation and muscle mass, and early differences in infant temperament^30^, increase the likelihood that females and males will adopt certain roles and behavioural characteristics^29^. Norms relating to sex differences in sensation-seeking might persist if sex differences in other traits ‘feed into' social norms that encourage males to challenge social conventions and discourage females from being open to potentially risky experiences. Cross-cultural studies could examine variation in self-reported sensation-seeking in relation to such norms; however, the SSS-V has been used in relatively few countries so far.

In summary, two ‘competing' interpretations of sex differences in personality have commonly been pitted against each other in the literature^31^,^32^: either males and females are differentially predisposed to exhibit particular personality traits, as a result of evolved psychological mechanisms; or males and females experience consistent social expectations across time periods that lead to the ‘channelling' of socially learned behaviour. We have discussed here how data from self-report traits of adults do not enable a clear distinction between these two approaches. However, ‘evolutionary' and ‘cultural' explanations of sex differences should not be seen as alterative explanations, and researchers are increasingly attempting to integrate these levels of explanation. For example, social role theorists have acknowledged a role for genetically influenced traits, with cultural processes either amplifying or countering evolved predispositions^15^. Similarly, evolutionary psychologists have called for more explicit recognition that cultural pressures can radically alter the cost-benefit analysis of certain behaviour patterns even when sex differences in predispositions exist^33^. Ultimately, a greater understanding of sex differences in personality scores will be gained by acknowledging that selection pressures act on both genetic and cultural inheritance pathways and that the interactions between these pathways during an individual's lifespan results in observed sex differences in behaviour^34^,^35^.

## Methods

### Literature review

We conducted an extensive literature review using the ISI Web of Science database with the search term ‘sensation seeking scale' (resulting in 573 articles since 1978), supplemented by cross-referencing within the literature and manual searches of references list of relevant articles and books. Relevant studies were those containing total SSS-V scores for males and females, and/or scores on one or more SSS-V sub-scales: TAS, Dis, BS and ES. Studies were then excluded where i) the sample size was smaller than 10 individuals per sex, ii) participants were younger than 17 years old, or iii) data were from populations selected on the basis of pathological, criminal or addictive behaviour. The values required from each study were means and SDs, or any measure of effect size (including t statistic, F ratio or correlation), and age of participants was also recorded. For those studies that reported relevant data without giving enough information to calculate effect sizes, the corresponding author was contacted; twenty-five out of seventy-seven authors replied with usable data (including one unpublished dataset). The majority of samples were undergraduates or community samples where the mean age was under 25. We restricted our main analyses to these samples to avoid confounding study year with participant age (see Table S2 in the Supplementary Information for samples with older participants and for data on variance ratios).

### Statistical analyses

For each dataset, we obtained the effect size Cohen's d, defined as the difference between male and female means divided by the pooled standard deviation. We used a random-effects model to obtain maximum likelihood estimates of overall effect sizes using SPSS macros^36^. A meta-regression was run with publication year as the predictor to examine change in effect sizes over time. We used the same method to examine whether scores for men and women have been changing over time. Tests for publication bias were conducted using a rank correlation between effect size and inverse variance and inspection of funnel plots. An alpha value of .05 was used throughout.

## Author Contributions

All authors contributed to developing the study concept and designing the study. Data collection was performed mainly by C.P.C. with assistance from D.C. and G.R.B. C.P.C. performed the data analysis and interpretation. C.P.C. and G.R.B. drafted the paper, and D.C. provided critical revisions. All authors approved the final version of the paper for submission.

## Supplementary Material

Supplementary Information

## Figures and Tables

**Table 1:** Effect sizes (d), 95% confidence intervals, sample sizes, number of male and female participants (N), and heterogeneity (Q) for total SSS-V scores and each sub-scale

Scale	d	95% CI	Samples	N_(Men)_	N_(Women)_	Heterogeneity (Q)
Total	0.46	0.41/0.51	67	7425	9511	53.73
TAS	0.42	0.37/0.46	68	7729	10356	63.47
Dis	0.46	0.41/0.52	67	7740	10155	54.19
BS	0.35	0.30/0.40	61	7162	9567	51.80
ES	0.04	−0.02/0.09	60	6952	9280	59.83
