# Supplementary material for: Sex differences in sensation-seeking: a meta-analysis
Source: Sci Rep. 2013 Aug 30;3:2486. doi: 10.1038/srep02486 (PMC3757272; doi:10.1038/srep02486)
Supplement: Supplementary Information [file srep02486-s1.pdf]

Supplementary information

Sex differences in sensation seeking: a meta-analysis

Catharine P. Cross[1], De-Laine M. Cyrenne[2], and Gillian R. Brown[1]

[1]University of St Andrews, UK

[2]California State University, Sacramento, USA

1) **Effect sizes for both age groups**
2) **List of all effect sizes retrieved**
3) **Men's and women's scores by study year**
4) **Funnel plots for all subscales**
5) **Variance Ratios**

1 – Effect sizes for both age groups

Sex differences on Total SSS-V, TAS, and Dis, are significantly larger in sample of older participants (Table ESM1, below). This is not really consistent with an evolutionary perspective (which would state that sexual selection on male competitiveness and risk-taking would peak in the late teens/early twenties, e.g. Wilson & Daly, 1985). It might be consistent with a social role interpretation (older participants have had more exposure to gendered social norms). However, neither interpretation should be made without caution for two reasons. First, the number of samples with older participants is relatively small and the age analysis is confounded with an undergraduate versus community sample distinction, making interpretation difficult (see Lipsey, 2003). Second, drawing inferences about individual-level variables (such as age) by using aggregate data risks misleading conclusions (Thompson & Higgins, 2002).

References

Lipsey, M. W. Those confounded moderators in meta-analysis: good, bad, and ugly. *Ann. Am. Acad. Politic. Soc. Sci.* **587,** 69-81 (2003).

Thompson, S. G. & Higgins, J. P. T. How should meta-regression analyses be undertaken and interpreted? *Statistics in Medicine*, **21,** 1559–73 (2002).

Wilson, M. & Daly, M. Competitiveness, risk taking, and violence: the young male syndrome. *Ethol. Sociobiol.* **73,** 59–73. (1985).

Table S1 Effect sizes by scale and age group

Age group	d	95% CI		k	N		Heterogeneity	Moderation
		LL	UL		Men	Women	(Q_{within})	($Q_{between}$)
				Total Sensation Seeking Scale				
Age <25	0.46	0.41	0.51	67	7425	9511	53.73	9.03**
Age >25	0.63	0.53	0.73	19	2299	2599	27.34	
**All samples**	**0.49**	**0.44**	**0.54**	**86**	**9724**	**12110**	**208.91*** **	
				Thrill and Adventure Seeking				
Age <25	0.42	0.37	0.46	68	7729	10356	63.47	16.63***
Age >25	0.60	0.52	0.67	25	2487	2682	17.87	
**All samples**	**0.46**	**0.42**	**0.50**	**95**	**11092**	**14037**	**170.97*** **	
				Disinhibition				
Age <25	0.46	0.41	0.52	67	7740	10155	54.19	3.92*
Age >25	0.57	0.48	0.66	27	2515	2659	43.45*	
**All samples**	**0.50**	**0.45**	**0.54**	**96**	**11131**	**13813**	**234.30*** **	
				Boredom Susceptibility				
Age <25	0.35	0.30	0.40	61	7162	9567	51.80	1.29
Age >25	0.38	0.29	0.47	23	2121	2316	19.97	
**All samples**	**0.34**	**0.30**	**0.49**	**87**	**10284**	**13145**	**172.08*** **	
				Experience Seeking				
Age <25	0.04	-0.02	0.09	60	6952	9280	59.83	0.06
Age >25	0.02	-0.07	0.12	25	2456	2622	21.77	
**All samples**	**0.02**	**-0.02**	**0.07**	**85**	**10284**	**12901**	**208.30*** **	

Note: Standard starring conventions apply.

2 – List of all effect sizes retrieved

Effect sizes are included in the main analysis if:

a) they are in age category 1 (mean age <25 OR sample described simply as ‘undergraduates’)

b) they are not identified as an outlier. Outliers were removed if they were more than 3 interquartile ranges above the upper quartile or more than 3 interquartile ranges below the lower quartile.

Table S2 All effect sizes retrieved by our search

Author(s)	Year	Subgroup	SSS-V Total d	NM	NF	Age	Region
Aluja et al	2004		0.45	367	639	1	3
Austin et al	2002		0.58	79	86	1	3
Ball et al	1984	20-29	0.3	110	103	1	3
Ball et al	1984	30-39	-0.17	86	67	2	3
Ball et al	1984	50-59	0.59	34	29	2	3
Ball et al	1984	40-49	0.27	54	44	2	3
Benjamin & Robbins	2007		0.34	24	48	1	1
Brocke et al	1999		2.3	14	18	1	3
Butkovic&Bratko	2003		0.85	118	148	2	3
Campbell et al	1993		0.74	34	20	2	1
Corulla	1988		0.23	58	134	1	3
de Vries et al	2009	Study 3	1	25	144	1	3
de Vries et al	2009	Study 1	0.52	52	252	1	3
Deckers&Ruch	1992		0.49	103	103	1	1
Dragutinovich	1987		0.75	106	105	1	3
Eckel& Grossman	2002		0.11	104	96	1	1
Eisenberg et al	2007		0.26	82	113	1	1
Eysenck and Haapasalo	1989		0.58	501	448	2	3
Fink et al	2006		0.7	120	158	1	3
Franken & Rowland	1990		0.5	113	142	1	1
Franken et al	1989		0.55	168	233	1	1
Franken et al	1994		0.58	121	179	1	1
Frantom& Sherman	1999		-0.21	20	34	2	1
Gabriel & Williamson	2010		0.32	77	73	1	1
Gilchrist et al	1995		0.52	36	57	2	3
Haapasalo	1990	20-29	0.57	107	98	1	3
Haapasalo	1990	30-39	0.45	123	100	2	3

		SSS-V Total					
**Author(s)**	**Year**	**Subgroup**	**d**	**NM**	**NF**	**Age**	**Region**
Haapasalo	1990	50-59	0.65	53	59	2	3
Haapasalo	1990	60-70	0.9	56	73	2	3
Haapasalo	1990	40-49	0.84	103	85	2	3
Hall	2005		0.68	55	118	1	1
Hampson et al	2008		0.41	87	77	1	1
Hanson et al	2008		0.27	16	12	1	1
Hromatko&Butkovic	2009		-0.02	150	51	1	3
Jack & Ronan	1998		0.74	36	15	1	3
Jaffe & Archer	1987		0.31	61	125	1	1
Joinson& Nettle	2005		0.61	125	323	2	3
Joireman et al	2002		0.78	40	66	1	1
Kang et al	2010		0.13	165	112	1	4
La Grange et al	1995		0.59	28	60	1	1
Labbe&Maisto	2008		0.34	234	250	1	1
Lang et al	2007		0.02	214	218	2	3
Lexington Longitudinal Database (Lynam, perscomm)	1996		0.64	438	570	1	1
McDaniel & Mahan	2008	students	0.37	113	88	1	1
McDaniel & Mahan	2008	non-students	0.67	125	131	2	1
McNamara & Ballard	1999		0.47	49	47	1	1
Mitchell	1999	non-smokers	0.66	10	10	1	1
Mitchell	1999	regular smokers	0.2	10	10	1	1
Nater et al	2005		0.71	26	27	2	3
O'Jile et al	2004		0.52	66	85	1	1
Ongen	2007		0.54	164	161	1	3
Perez &Torrubia	1986		0.62	50	83	1	3
Ridgeway & Russell	1980		0.34	155	181	1	1
Ripa et al	2001		0.79	363	328	2	3
Roberti	2004		-0.31	19	28	1	1
Rosenblitt et al	2001		0.48	68	75	1	1
Rowland & Franken	1986		0.55	299	439	1	1
Rowland et al	1989	Study 1	0.31	77	174	1	1
Rowland et al	1989	Study 2	0.44	84	168	1	1
Rowland et al	1988		0.65	20	20	1	1
Santelli et al	1990	US sample	0.1	25	36	1	1
Santelli et al	1990	Thai sample	0.16	45	116	1	4
Santelli et al	1990	Indian sample	0.54	103	100	1	4
Sasaki &Kanachi	2005		0.43	40	54	1	4
Satinder& Black	1984	cannabis nonusers	0.27	12	12	1	1
Satinder& Black	1984	cannabis users	0.08	12	12	1	1
Schroth	1990	high arousal	0.68	20	20	1	1

SSS-V Total							
Author(s)	Year	Subgroup	d	NM	NF	Age	Region
		condition					
Schroth	1990	low arousal condition	0.55	20	20	1	1
Schroth	1990	neutral arousal condition	0.62	20	20	1	1
Schroth	1995	Non-athletes	0.77	70	76	1	1
Schroth	1995	athletes	0.95	88	64	1	1
Schroth	1991	cohabiting couples	0.58	34	34	1	1
Schroth	1991	dating couples	0.66	34	34	1	1
Schroth	1991	married couples	1.28	34	34	2	1
Steenkamp& Baumgartner	1992		0.55	112	112	1	3
Terasaki&Imada	1988		0.13	66	39	1	4
Tonetti et al	2010		0.35	408	633	1	3
van Wijk	2007		0.51	656	202	1	3
Voracek et al	2010		0.49	84	114	2	3
Vuust et al	2010		0.28	59	64	1	3
Watt & Ewing	1996		0.54	84	169	1	1
Zacny	2010		0.17	51	47	1	1
Zuckerman &Litle	1986		0.41	213	89	1	1
Zuckerman &Neeb	1980	1980 study	0.27	331	554	1	1
Zuckerman &Neeb	1980	existing norms	0.49	337	646	1	1
Zuckerman et al	1978	college students	0.32	97	122	1	1
Zuckerman et al	1978	20-29	0.6	119	250	1	3
Zuckerman et al	1978	30-39	0.95	25	145	2	1
Zuckerman et al	1978	40-49	0.83	26	89	2	3
Zuckerman et al	1991		0.71	178	347	1	1

TAS subscale							
Authors	Year	Subgroup	d	NM	NF	Age	Region
Aluja et al	2004		0.4	367	639	1	0
Austin et al	2002		0.31	79	86	1	3
Ball et al	1984	20-29	0.43	110	103	1	3
Ball et al	1984	50-59	0.49	34	29	2	3
Ball et al	1984	30-39	0.44	86	67	2	3
Ball et al	1984	40-49	0.47	54	44	2	3
Beck et al	1995	high intensity drinkers	0.54	168	108	1	1
Beck et al	1995	low intensity drinkers	0.41	185	320	1	1
Benjamin & Robbins	2007		0.3	24	48	1	1
Bjork et al	2004		0.34	27	14	2	1

		TAS subscale					
**Authors**	**Year**	**Subgroup**	**d**	**NM**	**NF**	**Age**	**Region**
Brocke et al	1999		0.95	14	18	1	3
Butkovic&Bratko	2003		0.62	118	148	2	3
Campbell et al	1993		1.13	34	20	2	1
Casillas	2006		0.49	84	125	2	1
Corulla	1988		0.27	58	134	1	3
Curran	2006		-0.35	61	19	2	1
de Vries et al	2009	Study 1	0.57	52	252	1	3
de Vries et al	2009	Study 3	0.71	25	144	1	3
Deckers&Ruch	1992		0.39	103	103	1	1
Demaree et al	2008		0.48	32	27	1	1
Dragutinovich	1987		0.5	106	105	1	3
Eckel& Grossman	2002		0.04	104	96	1	1
Eisenberg et al	2007		0.17	82	113	1	1
Eysenck and Haapasalo	1989		0.52	501	448	2	3
Fein, Sclafani, and Finn	2010		0.34	53	65	1	1
Fink et al	2006		0.26	120	158	1	3
Flory et al	2006		0.44	154	197	2	1
Franken et al	1994		0.54	121	179	1	1
Frantom& Sherman	1999		-0.11	20	34	2	1
Gilchrist et al	1995		0.51	36	57	2	3
Glickson&Abulafia	1998	29-32	0.27	21	15	2	5
Glickson&Abulafia	1998	17-20	0.8	39	23	1	5
Glickson&Abulafia	1998	25-28	0.7	89	77	2	5
Glickson&Abulafia	1998	21-24	0.45	85	166	1	5
Glickson&Abulafia	1998	33-36	1.05	27	19	2	5
Glickson&Abulafia	1998	41-50	0.51	19	52	2	5
Glickson&Abulafia	1998	51-60	0.43	10	17	2	5
Haapasalo	1990	20-29	0.73	107	98	1	3
Haapasalo	1990	30-39	0.46	123	100	2	3
Haapasalo	1990	40-49	0.83	103	85	2	3
Haapasalo	1990	50-59	0.62	53	59	2	3
Haapasalo	1990	60-70	0.68	56	73	2	3
Hampson et al	2008		0.49	87	77	1	1
Hanson et al	2008		0.76	16	12	1	1
Hromatko&Butkovic	2009		0.04	150	51	1	3
Jack & Ronan	1998		0.27	36	15	1	3
Joinson& Nettle	2005		0.47	125	323	2	3
Joireman et al	2002		0.56	42	69	1	1
Justus et al	2001		0.41	87	103	1	1
Kang et al	2010		0.24	165	112	1	4
La Grange et al	1995		0.65	28	60	1	1
Labbe&Maisto	2008		0.25	248	260	1	1

TAS subscale							
**Authors**	**Year**	**Subgroup**	**d**	**NM**	**NF**	**Age**	**Region**
Lexington Longitudinal Database (Lynam, perscomm)	1996		0.59	438	570	1	1
Litman et al	2005		0.39	150	402	1	1
Lundahl	1995		1.2	21	23	1	1
McDaniel & Mahan	2008	non-students	0.76	125	131	2	1
McDaniel & Mahan	2008	students	0.25	113	88	1	1
Mergl et al	2006		0.75	20	26	2	3
Mitchell	1999	non-smokers	0.65	10	10	1	0
Mitchell	1999	regular smokers	0.24	10	10	1	0
Nater et al	2005		0.44	26	27	2	3
(Mergl et al)**	2003		0.56	703	823		3
O'Jile et al	2004		0.56	66	85	1	1
Ongen	2007		0.29	164	161	1	3
Perez &Torrubia	1986		0.24	50	83	1	3
Perez &Torrubia	1985		0.26	173	176		3
Pfefferbaum et al	1994		0.54	148	148	1	1
Rahmani&Lavasani	2012		0.43	50	83	1	5
Rawlings	2003		0.42	40	160	1	3
Rawlings Vidal &Furnham	2000	Barcelona sample	0.12	15	60	1	3
Rawlings Vidal &Furnham	2000	London sample	0.25	17	62	1	3
Rawlings*	2003*		0.44	11	69	1	3
Ripa et al	2001		0.8	363	328	2	3
Roberti	2004		0.38	19	28	1	1
Rosenblitt et al	2001		0.53	68	75	1	1
Rowland & Franken	1986		0.52	299	439	1	1
Satinder& Black	1984	cannabis nonusers	1.23	12	12	1	1
Satinder& Black	1984	cannabis users	0.4	12	12	1	1
Schroth	1995	athletes	0.66	88	64	1	1
Schroth	1995	non-athletes	0.56	70	76	1	1
Schroth	1991	cohabiting couples	0.45	34	34	1	1
Schroth	1991	dating couples	0.74	34	34	1	1
Schroth	1991	married couples	1.02	34	34	2	1
Schroth	1990	high arousal condition	0.65	20	20	1	1
Schroth	1990	low arousal condition	0.45	20	20	1	1
Schroth	1990	neutral arousal condition	0.55	20	20	1	1

TAS subscale							
**Authors**	**Year**	**Subgroup**	**d**	**NM**	**NF**	**Age**	**Region**
Terasaki&Imada	1988		0.29	66	39	1	4
Thornquist et al	1991		0.82	55	55	1	1
Tonetti et al	2010		0.26	408	633	1	3
Torrubia et al	2001		0.13	229	599	1	3
van Wijk	2007		0.59	656	202	1	3
Voracek et al	2010		0.61	84	114	2	3
Vuust et al	2010		0.2	59	64	1	3
Watt & Ewing	1996		0.63	84	169	1	1
Zacny	2010		0	51	47	1	1
Zuckerman &Litle	1986		0.58	213	89	1	1
Zuckerman &Neeb	1980	1980 study	0.42	331	554	1	1
Zuckerman &Neeb	1980	existing norms	0.3	337	646	1	1
Zuckerman et al	1978	college students	0.36	97	122	1	1
Zuckerman et al	1988		0.54	73	198	1	1
Zuckerman et al	1991		0.51	178	347	1	1

Disinhibition							
**Authors**	**Year**	**Subgroup**	**d**	**NM**	**NF**	**Age**	**Region**
Aluja et al	2004		0.56	367	639	1	3
Austin et al	2002		0.68	79	86	1	3
Ball et al	1984	20-29	0.29	110	103	1	3
Ball et al	1984	40-49	0.23	54	44	2	3
Ball et al	1984	30-39	-0.29	86	67	2	0
Ball et al	1984	50-59	0.58	34	29	2	3
Beck et al	1995	high intensity drinkers	0.62	168	108	1	1
Beck et al	1995	low intensity drinkers	0.39	185	320	1	1
Benjamin & Robbins	2007		0.2	24	48	1	1
Bjork et al	2004		0.48	27	14	2	1
Brocke et al	1999		0.96	14	18	1	3
Butkovic&Bratko	2003		0.74	118	148	2	3
Campbell et al	1993		0.63	34	20	2	1
Casillas	2006		0.72	84	125	2	1
Corulla	1988		-0.06	58	134	1	3
Curran	2006		-0.27	61	19	2	1
de Vries et al	2009	Study 1	0.22	52	252	1	3
de Vries et al	2009	Study 3	1.25	25	144	1	3
Deckers&Ruch	1992		0.47	103	103	1	1
Diehm&Armatas	2004		0.69	59	26	2	3
Dragutinovich	1987		0.71	106	105	1	3
Eckel& Grossman	2002		0.33	104	96	1	1
Eisenberg et al	2007		0.16	82	113	1	1

Authors	Year	Subgroup	Disinhibition d	NM	NF	Age	Region
Eysenck and Haapasalo	1989		0.73	501	448	2	3
Fein et al	2010		0.65	53	65	2	1
Fink et al	2006		0.63	120	158	1	3
Flory et al	2006		0.76	154	197	2	1
Franken et al	1994		0.62	121	179	1	1
Frantom& Sherman	1999		-0.22	20	34	2	1
Gilchrist et al	1995		0.47	36	57	2	3
Glickson&Abulafia	1998	21-24	0.31	85	166	1	5
Glickson&Abulafia	1998	17-20	0.5	39	23	1	5
Glickson&Abulafia	1998	33-36	0.08	27	19	2	5
Glickson&Abulafia	1998	25-28	0.33	89	77	2	5
Glickson&Abulafia	1998	29-32	1.12	21	15	2	5
Glickson&Abulafia	1998	51-60	0.58	10	17	2	5
Glickson&Abulafia	1998	41-50	0.77	19	52	2	5
Haapasalo	1990	20-29	0.58	107	98	1	3
Haapasalo	1990	30-39	0.56	123	100	2	3
Haapasalo	1990	40-49	0.88	103	85	2	3
Haapasalo	1990	50-59	0.72	53	59	2	3
Haapasalo	1990	60-70	1.03	56	73	2	3
Hampson et al	2008		0.33	87	77	1	1
Hanson	2008		0.37	16	12	1	1
Hromatko&Butkovic	2009		0.56	150	51	1	3
Jack & Ronan	1998		0.81	36	15	1	3
Joinson& Nettle	2005		0.51	125	323	2	3
Joireman et al	2002		0.71	39	68	1	1
Justus et al	2001		0.41	87	103	1	1
Kang et al	2010		0.16	165	112	1	4
La Grange et al	1995		0.39	28	60	1	1
Labbe&Maisto	2008		0.43	245	254	1	1
Lexington Longitudinal Database (Lynam, perscomm)	1996		0.41	438	570	1	1
McDaniel & Mahan	2008	non-students	0.68	125	131	2	1
McDaniel & Mahan	2008	students	0.42	113	88	1	1
Mergl et al	2006		0.54	20	26	2	3
Mitchell	1999	non-smokers	0.06	10	10	1	1
Mitchell	1999	regular smokers	0.51	10	10	1	1
Nater et al	2005		0.52	26	27	2	3
(Mergl et al)**	2003		0.45	703	823		3
O'Jile et al	2004		0.41	66	85	1	1
Ongen	2007		0.75	164	161	1	3
Perez &Torrubia	1986		0.95	50	83	1	3
Perez &Torrubia	1985		0.94	173	176		3

Authors	Year	Subgroup	d	NM	NF	Age	Region
		**Disinhibition**					
Perkins et al	2008		0.6	51	80	1	1
Rahmani&Lavasani	2012		0.33	69	108	1	5
Rawlings	2003		0.35	40	160	1	3
Rawlings et al	2000	Barcelona sample	0.33	15	60	1	3
Rawlings et al	2000	London sample	0.42	17	62	1	3
Rawlings	2003*		0.52	11	69	1	3
Ripa et al	2001		0.61	363	328	2	3
Roberti	2004		-0.03	19	28	1	1
Rosenblitt et al	2001		0.25	68	75	1	1
Rowland & Franken	1986		0.43	299	439	1	1
Satinder& Black	1984	cannabis nonusers	0.18	12	12	1	1
Satinder& Black	1984	cannabis users	0.25	12	12	1	1
Schroth	1995	athletes	0.87	88	64	1	1
Schroth	1995	Non-athletes	0.72	70	76	1	1
Schroth	1991	cohabiting couples	0.61	34	34	1	1
Schroth	1991	dating couples	0.48	34	34	1	1
Schroth	1991	married couples	1.58	34	34	2	1
Schroth	1990	high arousal condition	0.88	20	20	1	1
Schroth	1990	low arousal condition	0.68	20	20	1	1
Schroth	1990	neutral arousal condition	0.7	20	20	1	1
Terasaki&Imada	1988		0	66	39	1	4
Tonetti et al	2010		0.41	408	633	1	3
Torrubia et al	2001		0.72	229	599	1	3
van Wijk	2007		0.46	656	202	1	3
Vuust et al	2010		0.22	59	64	1	3
Watt & Ewing	1996		0.47	84	169	1	1
White & Johnson	1988		0.72	203	213	1	1
Zacny	2010		0.49	51	47	1	1
Zuckerman &Litle	1986		0.29	213	89	1	1
Zuckerman &Neeb	1980	actual study	0.24	331	554	1	1
Zuckerman &Neeb	1980	norms	0.49	337	646	1	1
Zuckerman et al	1978	college students	0.45	97	122	1	1
Zuckerman et al	1988		0.29	73	198	1	1
Zuckerman et al	1991		0.58	178	347	1	1

		Boredom Susceptibility					
**Authors**	**Year**	**Subgroup**	**d**	**NM**	**NF**	**Age**	**Region**
Aluja et al	2004		0.22	367	639	1	3
Austin et al	2002		0.45	79	86	1	3
Ball et al	1984	20-29	0.25	110	103	1	3
Ball et al	1984	30-39	-0.05	86	67	2	3
Ball et al	1984	40-49	0.38	54	44	2	3
Ball et al	1984	50-59	0.58	34	29	2	3
Benjamin & Robbins	2007		0.71	24	48	1	1
Bjork et al	2004		0.6	27	14	2	1
Brocke et al	1999		1.23	14	18	1	3
Butkovic&Bratko	2003		0.73	118	148	2	3
Campbell et al	1993		-0.01	34	20	2	1
Casillas	2006		0.32	84	125	2	1
Corulla	1988		0.33	58	134	1	3
Curran	2006		-0.43	61	19	2	1
de Vries et al	2009	Study 1	0.33	52	252	1	3
de Vries et al	2009	Study 3	0.65	25	144	1	3
Deckers&Ruch	1992		0.37	103	103	1	1
Demaree et al	2008		0.1	32	27	1	1
Dragutinovich	1987		0.67	106	105	1	3
Eckel& Grossman	2002		0.29	104	96	1	1
Eisenberg et al	2007		0.29	82	113	1	1
Eysenck&Haapasalo	1989		0.16	501	448	2	3
Fein et al	2010		0.54	53	65	2	1
Fink et al	2006		0.61	120	158	1	3
Flora	2007		-0.08	125	263		1
Flory et al	2006		0.4	154	197	2	1
Ford	1995		0	220	252	1	1
Franken	1994		0.44	121	179	1	1
Frantom& Sherman	1999		0.24	20	34	2	1
Gilchrist et al	1995		0.4	36	57	2	3
Glickson&Abulafia	1998	17-20	-0.25	39	23	1	5
Glickson&Abulafia	1998	21-24	0.29	85	166	1	5
Glickson&Abulafia	1998	33-36	0.24	27	19	2	5
Glickson&Abulafia	1998	25-28	0.32	89	77	2	5
Glickson&Abulafia	1998	29-32	0.72	21	15	2	5
Glickson&Abulafia	1998	41-50	0.13	19	52	2	5
Glickson&Abulafia	1998	51-60	0.88	10	17	2	5
Hampson et al	2008		0.24	87	77	1	1
Hanson	2008		0.36	16	12	1	1
Hromatko&Butkovic	2009		0.19	150	51	1	3
Jack & Ronan	1998		0.6	36	15	1	3
Joinson& Nettle	2005		0.53	125	323	2	3
Joireman et al	2002		0.6	42	69	1	1
Justus et al	2001		0.37	87	103	1	1

Authors	Year	Subgroup	d	NM	NF	Age	Region
		**Boredom Susceptibility**					
Kang et al	2010		0.11	165	112	1	4
La Grange et al	1995		0.2	28	60	1	1
Labbe&Maisto	2008		0.32	240	260	1	1
Lexington Longitudinal Database (Lynam, perscomm)	1996		0.59	438	570	1	1
Lundahl	1995		0.66	21	23	1	1
McDaniel & Mahan	2008	non-students	0.35	125	131	2	1
McDaniel & Mahan	2008	students	0.47	113	88	1	1
Mergl et al	2006		0.61	20	26	2	3
Mitchell	1999	non-smokers	0.9	10	10	1	0
Mitchell	1999	regular smokers	0	10	10	1	0
Nater et al	2005		0.41	26	27	2	3
(Mergl et al)**	2003		0.16	703	823		3
O'Jile et al	2004		0.37	66	85	1	1
Ongen	2007		0.31	164	161	1	3
Perez &Torrubia	1986		0.28	50	83	1	3
Perez &Torrubia	1985		0.3	173	176		3
Rahmani&Lavasani	2012		0.36	69	108	1	5
Rawlings	2003		0.01	40	160	1	3
Rawlings et al	2000	Barcelona sample	0.72	15	60	1	3
Rawlings et al	2000	London sample	-0.38	17	62	1	3
Rawlings	2003*		0.31	11	69	1	3
Ripa et al	2001		0.48	363	328	2	3
Roberti	2004		-0.29	19	28	1	1
Rosenblitt et al	2001		0.3	68	75	1	1
Rowland & Franken	1986		0.45	299	439	1	1
Satinder & Black	1984	cannabis nonusers	0	12	12	1	1
Satinder & Black	1984	cannabis users	-0.14	12	12	1	1
Schroth	1995	athletes	0.7	88	64	1	1
Schroth	1995	Non-athletes	0.77	70	76	1	1
Schroth	1991	cohabiting couples	0.51	34	34	1	1
Schroth	1991	dating couples	0.49	34	34	1	1
Schroth	1991	married couples	0.27	34	34	2	1
Schroth	1990	high arousal condition	0.48	20	20	1	1
Schroth	1990	low arousal condition	0.3	20	20	1	1
Schroth	1990	neutral arousal	0.65	20	20	1	1

Boredom Susceptibility							
**Authors**	**Year**	**Subgroup**	**d**	**NM**	**NF**	**Age**	**Region**
Terasaki&Imada	1988	condition	0.55	66	39	1	4
Tonetti et al	2010		0.32	408	633	1	3
Torrubia et al	2001		0.31	229	599	1	3
van Wijk	2007		0.17	656	202	1	3
Vodanovich&Kass	1990		0.5	68	130	1	1
Vuust et al	2010		0.3	59	64	1	3
Watt & Ewing	1996		0.39	84	169	1	1
Zacny	2010		0.27	51	47	1	1
Zuckerman &Litle	1986		0.32	213	89	1	1
Zuckerman &Neeb	1980	actual study	0.08	331	554	1	1
Zuckerman &Neeb	1980	norms	0.39	337	646	1	1
Zuckerman et al	1978	college students	0.1	97	122	1	1
Zuckerman et al	1988		0.25	73	198	1	1
Zuckerman et al	1991		0.49	178	347	1	1

Experience Seeking							
**Authors**	**Year**	**Subgroup**	**d**	**NM**	**NF**	**Age**	**Region**
Aluja et al	2004		0.02	367	639	1	3
Austin et al	2002		0.05	79	86	1	3
Ball et al	1984	20-29	-0.28	110	103	1	3
Ball et al	1984	30-39	-0.68	86	67	2	3
Ball et al	1984	40-49	-0.25	54	44	2	3
Ball et al	1984	50-59	0.22	34	29	2	3
Benjamin & Robbins	2007		-0.18	24	48	1	1
Bjork et al	2004		0.14	27	14	2	1
Brocke et al	1999		0.93	14	18	1	3
Butkovic&Bratko	2003		0.22	118	148	2	3
Campbell et al	1993		0.09	34	20	2	1
Corulla	1988		0.09	58	134	1	3
Curran	2006		-0.6	61	19	2	1
de Vries et al	2009	Study 1	0.46	52	252	1	3
de Vries et al	2009	Study 3	1.06	25	144	1	3
Deckers&Ruch	1992		0.12	103	103	1	1
Demaree et al	2008		0.39	32	27	1	1
Eckel& Grossman	2002		-0.42	104	96	1	1
Eisenberg et al	2007		0.02	82	113	1	1
Eysenck and Haapasalo	1989		-0.15	501	448	2	3
Fein et al	2010		-0.13	53	65	2	1
Fink et al	2006		0.28	120	158	1	3
Flory et al	2006		0.19	154	197	2	1
Franken et al	1994		-0.11	121	179	1	1

Experience Seeking							
**Authors**	**Year**	**Subgroup**	**d**	**NM**	**NF**	**Age**	**Region**
Frantom& Sherman	1999		-0.54	20	34	2	1
Gilchrist et al	1995		0.02	36	57	2	3
Glickson&Abulafia	1998	17-20	0	39	23	1	5
Glickson&Abulafia	1998	21-24	-0.09	85	166	1	5
Glickson&Abulafia	1998	51-60	0.82	10	17	2	5
Glickson&Abulafia	1998	33-36	0.07	27	19	2	5
Glickson&Abulafia	1998	29-32	0.16	21	15	2	5
Glickson&Abulafia	1998	25-28	0.31	89	77	2	5
Glickson&Abulafia	1998	41-50	0.07	19	52	2	5
Haapasalo	1990	20-29	-0.35	107	98	1	3
Haapasalo	1990	30-39	-0.29	123	100	2	3
Haapasalo	1990	40-49	-0.07	103	85	2	3
Haapasalo	1990	50-59	-0.24	53	59	2	3
Haapasalo	1990	60-70	0.06	56	73	2	3
Hampson et al	2008		0.05	87	77	1	1
Hanson et al	2008		-0.71	16	12	1	1
Hromatko&Butkovic	2009		-0.88	150	51	1	3
Jack & Ronan	1998		0.25	36	15	1	3
Joinson& Nettle	2005		0.16	125	323	2	3
Joireman et al	2002		0.48	41	67	1	1
Kang et al	2010		-0.19	165	112	1	4
La Grange et al	1995		0.18	28	60	1	1
Labbe&Maisto	2008		-0.14	244	260	1	1
Lexington Longitudinal Database (Lynam, perscomm)	1996		0.2	438	570	1	1
Litman et al	2005		0.09	150	402	1	1
McDaniel & Mahan	2008	non-students	0.15	125	131	2	1
McDaniel & Mahan	2008	students	-0.07	113	88	1	1
McNamara & Ballard	1999		0.4	49	47	1	1
Mergl et al	2006		0.45	20	26	2	3
Mitchell	1999	non-smokers	-0.52	10	10	1	0
Mitchell	1999	regular smokers	-0.37	10	10	1	0
Nater et al	2005		0.27	26	27	2	3
(Mergl et al)**	2003		0.05	703	823		3
Ongen	2007		0.19	164	161	1	3
Perez &Torrubia	1986		0.1	50	83	1	3
Perez &Torrubia	1985		-0.2	173	176		3
Perkins et al	2008		0.25	51	80	1	1
Rahmani&Lavasani	2012		0.27	69	108	1	5
Rawlings	2003		0.07	40	160	1	3
Rawlings et al	2000	Barcelona	0.5	15	60	1	3

Experience Seeking							
**Authors**	**Year**	**Subgroup**	**d**	**NM**	**NF**	**Age**	**Region**
Rawlings et al	2000	London sample	0.03	17	62	1	3
Rawlings	2003*		0.31	11	69	1	3
Ripa et al	2001		0.12	363	328	2	3
Roberti	2004		-1.23	19	28	1	1
Rosenblitt et al	2001		0.3	68	75	1	1
Rowland & Franken	1986		0.59	299	439	1	1
Satinder& Black	1984	cannabis nonusers	-0.56	12	12	1	1
Satinder& Black	1984	cannabis users	-0.22	12	12	1	1
Schroth	1995	athletes	0.1	88	64	1	1
Schroth	1995	Non-athletes	-0.1	70	76	1	1
Schroth	1991	cohabiting couples	-0.06	34	34	1	1
Schroth	1991	dating couples	-0.1	34	34	1	1
Schroth	1991	married couples	0.79	34	34	2	1
Schroth	1990	high arousal condition	-0.16	20	20	1	1
Schroth	1990	low arousal condition	0	20	20	1	1
Schroth	1990	neutral arousal condition	-0.3	20	20	1	1
Terasaki&Imada	1988		-0.39	66	39	1	4
Torrubia et al	2001		0.01	229	599	1	3
van Wijk	2007		-0.01	656	202	1	3
Voracek et al	2010		0.05	84	114	2	3
Vuust et al	2010		-0.08	59	64	1	3
Watt & Ewing	1996		0.04	84	169	1	1
White & Johnson	1988		0.07	203	211	1	1
Zacny	2010		-0.3	51	47	1	1
Zuckerman &Litle	1986		-0.06	213	89	1	1
Zuckerman &Neeb	1980	norms	0	337	646	1	1
Zuckerman &Neeb	1980	actual study	0	331	554	1	1
Zuckerman et al	1978	college students	-0.1	97	122	1	1
Zuckerman et al	1988		-0.04	73	198	1	1
Zuckerman et al	1991		0.18	178	347	1	1

* Unpublished ** Norms reported here from a different paper.

d = standardised sex difference. NM: Number of men. NF: Number of women. Region: 1 = North America; 2 = South America; 3 = Europe, Australia, New Zealand, South Africa; 4 =

Asia; 5 = Middle East. Age: 1 = Mean age <25 years OR sample identified simply as 'undergraduates'; 2 = mean age > 25; 0 = information not available.

References used in meta-analysis

Aluja, A., Garcia, O. & Garcia, L. F. Exploring the structure of Zuckerman's sensation seeking scale, Form V in a Spanish sample. *Psych. Rep.* **95,** 338-344 (2004).

Austin, E. J., Manning, J. T., McInroy, K. & Mathews, E. A preliminary investigation of the associations between personality, cognitive ability and digit ratio. *Pers. Indiv. Diff.* **33,** 1115-1124 (2002).

Ball, I. L., Farnill, D. & Wangeman, J. F. Sex and age-differences in sensation seeking: Some national comparisons. *Brit. J. Psychol.* **75,** 257-265 (1984).

Beck, K. H., Thombs, D. L., Mahoney, A. & Fingar, K. M. Social context and sensation seeking: Gender differences in college-student drinking motivations. *Int. J. Addic.* ***30,*** 1101-1115 (1995).

Benjamin, A. M. & Robbins, S. J. The role of framing effects in performance on the Balloon Analogue Risk Task (BART). *Pers. Individ. Diff.* **43,** 221-230 (2007).

Bjork, J. M., Hommer, D. W., Grant, S. J. & Danube, C. Impulsivity in abstinent alcohol-dependent patients: relation to control subjects and type 1-/type 2-like traits. *Alcohol* ***34,*** 133-150 (2004).

Brocke, B., Beauducel, A. & Tasche, K. G. Biopsychological bases and behavioral correlates of sensation seeking: contributions to a multilevel validation. *Pers. Indiv. Diff.* ***26,*** 1103-1123 (1999).

Butkovic, A. & Bratko, D. Generation and sex differences in sensation seeking: Results of the family study. *Percep. Motor Skills* **97,** 965-970 (2003).

Campbell, J. B., Tyrrell, D. J. & Zingaro, M. Sensation seeking among white-water canoe and kayak paddlers. *Personality and Individual Differences,* **14,** 489-491 (1993).

Casillas. Personality and neuropsychological correlates of impulsivity. *Dissertation Abstracts International: Section B: The Sciences and Engineering, 66, (8-B),* 4476 (2006).

Corulla, W. J. A further psychometric investigation of the Sensation Seeking Scale Form V and its relationship to the EPQ-R and the I7 Impulsiveness Questionnaire. *Personality and Individual Differences,* **9,** 277-287 (1988).

Curran, M. F. Risk taking as a contributing factor to driving while under the influence of alcohol. *Dissertation Abstracts International: Section B: The Sciences and Engineering, 67, (3-B),* 1741 (2006).

de Vries, R. E., de Vries, A. & Feij, J. A. Sensation seeking, risk-taking, and the HEXACO model of personality. *Personality and Individual Differences,* **47,** 536-540 (2009).

Deckers, L. & Ruch, W. Sensation seeking and the Situational Humor Response Questionnaire (SHRQ): its relationship in American and German samples. *Personality and Individual Differences,* **13,** 1051-1054 (1992).

Demaree, H. A., DeDonno, M. A., Burns, K. J. & Everhart, D. E. You bet: How personality differences affect risk-taking preferences. *Pers. Indiv. Diff.* **44,** 1484-1494 (2008).

Diehm, R., & Armatas, C. Surfing: an avenue for socially acceptable risk-taking, satisfying needs for sensation seeking and experience seeking. *Pers. Indiv. Diff.* **36,** 663-677 (2004).

Dragutinovich, S. Stimulus-intensity reducers: Are they sensation seekers, extroverts, and strong nervous types? *Pers. Indiv. Diff.* **8,** 693-704 (1987).

Eckel, C. C. & Grossman, P. J. (2002). Sex differences and statistical stereotyping in attitudes toward financial risk. *Evolution and Human Behavior* **23,** 281-295 (2002).

Eisenberg, D. T. A., Campbell, B., MacKillop, J., Lum, J. K. & Wilson, D. S. Season of birth and dopamine receptor gene associations with impulsivity, sensation seeking and reproductive behaviors. *Plos One*, **2,** doi: 10.1371/journal.pone.0001216 (2007).

Eysenck, S. B. G. & Haapasalo, J. Cross-cultural comparisons of personality: Finland and England. *Pers. Indiv. Diff.* **10,** 121-125 (1989).

Fein, G., Di Sclafani, V., & Finn, P. Sensation seeking in long-term abstinent alcoholics, treatment-naive active alcoholics, and nonalcoholic controls. *Alcoholism – Clin. Exp. Res.* **34,** 1045-1051 (2010).

Fink, B., Neave, N., Laughton, K., & Manning, J. T. Second to fourth digit ratio and sensation seeking. *Pers. Indiv. Diff.* **41,** 1253-1262 (2006).

Flora, R. L. Behavioral undercontrol and alcohol consumption. *Dissertation Abstracts International: Section B: The Sciences and Engineering, 68, (1-B),* 620 (2007).

Flory, J. D. *et al.* Dispositional impulsivity in normal and abnormal samples. *J. Psychiatric Res.* **40,** 438-447 (2006).

Ford, C. M. Psychosocial correlates of contraceptive and HIV/STD preventive behaviors among college students. *Dissertation Abstracts International: Section B: The Sciences and Engineering, 55, (10-B),* 4590 (1995).

Franken, R. E., Gibson, K. & Rowland, G. L. Sensation seeking and feelings about the forced-choice format. *Pers. Indiv. Diff.* **10,** 337-339 (1989).

Franken, R. E., Hill, R. & Kierstead, J. Sport interest as predicted by the personality measures of competitiveness, mastery, instrumentality, expressivity, and sensation seeking. *Pers. Indiv. Diff.* **17,** 467-476 (1994)..

Franken, R. E. & Rowland, G. L. Sensation seeking and fantasy. *Pers. Indiv. Diff.* **11,** 191-193 (1990).

Frantom, C., & Sherman, M. F. At what price art? Affective instability within a visual art population. *Creativity Research Journal* **12,** 15-23 (1999).

Gabriel, K. I. & Williamson, A. Framing alters risk-taking behavior on a modified Balloon Analogue Risk Task (BART) in a sex-specific manner. *Psych. Rep. 107,* 699-712 (2010).

Gilchrist, H., Povey, R., & Dickinson, A. The Sensation Seeking Scale: Its use in a study of the characteristics of people choosing adventure holidays. *Pers. Indiv. Diff.* **19,** 513-51 (1995).

Glicksohn, J., & Abulafia, J. Embedding sensation seeking within the big three. *Pers. Indiv. Diff.* **25,** 1085-1099(1998).

Haapasalo, J. The Eysenck Personality Questionnaire and Zuckerman Sensation Seeking Scale (Form V) in Finland: Age differences. *Pers. Indiv. Diff.* **11,** 503-508 (1990).

Hall, A. Sensation seeking and the use and selection of media materials. *Psychological Reports,* **97,** 236-244 (2005).

Hampson, E., Ellis, C. L. & Tenk, C. M. On the relation between 2D : 4D and sex-dimorphic personality traits. *Arch. Sex. Behav.* **37,** 133-144 (2008).

Hanson, K. L., Luciana, M. & Sullwold, K. Reward-related decision-making deficits and elevated impulsivity among MDMA and other drug users. *Drug Alcohol Depend.* **96,** 99-110(2008).

Hromatko, I. & Butkovic, A. Sensation seeking and spatial ability in athletes: an evolutionary account. *Journal of Human Kinetics,* **21,** 5-13 (2009).

Jack, S. J. & Ronan, K. R. Sensation seeking among high- and low-risk sports participants. *Pers. Indiv. Diff.* **25,** 1063-1083 (1998).

Jaffe, L. T. & Archer, R. P. The prediction of drug-use among college-students from MMPI, MCMI, and Sensation Seeking Scales. *J. Pers. Assess.* **51,** 243-253 (1987).

Joinson, C. & Nettle, D. Season of birth variation in sensation seeking in an adult population. *Pers. Indiv. Diff.* ***38,*** 859-870 (2005).

Joireman, J. A., Fick, C. S. & Anderson, J. W. Sensation seeking and involvement in chess. *Pers. Indiv. Diff.* **32,** 509-515 (2002).

Justus, A. N., Finn, P. R. & Steinmetz, J. E. P300, disinhibited personality, and early-onset alcohol problems. *Alcoholism – Clin. Exp. Res.* **25,** 1457-1466 (2001).

Kang, J. I., Song, D.-H., Namkoong, K. & Kim, S. J. Interaction effects between COMT and BDNF polymorphisms on boredom susceptibility of sensation seeking traits. *Psychiat. Res.* **178,** 132-136 (2010).

Labbe, A. K. & Maisto, S. A. Heavy alcohol use and other predictors of non-prescribed use of prescription stimulant medication among college students. *Alcoholism – Clin. Exp. Res.* **32,** 181A-181A (2008).

Lagrange, L., Jones, T. D., Erb, L. & Reyes, E. Alcohol consumption: Biochemical and personality correlates in a college student population. *Addictive Behav.* **20,** 93-103 (1995).

Lang, U. E., Bajbouj, M., Sander, T. & Gallinat, J. Gender-dependent association of the functional catechol-O-methyltransferase Val158Met genotype with sensation seeking personality trait. *Neuropsychopharmacol. 32,* 1950-1955 (2007).

Litman, J. A., Collins, R. P. & Spielberger, C. D. The nature and measurement of sensory curiosity. *Pers. Indiv. Diff.* **39,** 1123-1133 (2005).

Lundahl, L. H. Separation of the effects of family history of alcoholism, heavy drinking, and gender on personality functioning and sensitivity to alcohol in college students. *Dissertation Abstracts International: Section B: The Sciences and Engineering,* **56,** 2874 (1995).

McDaniel, S. R. & Mahan, J. E., III. An examination of the ImpSS scale as a valid and reliable alternative to the SSS-V in optimum stimulation level research. *Pers. Indiv. Diff.* **44,** 1528-1538 (2008).

McNamara, L., & Ballard, M. E. Resting arousal, sensation seeking, and music preference. *Genetic Social and General Psychology Monographs* ***125,*** 229-250 (1999).

Mergl, R. *et al.* Facial expressions and personality: a kinematical investigation during an emotion induction experiment. *Neuropsychobiol.* **54,** 114-119 (2006).

Mitchell, S. H. Measures of impulsivity in cigarette smokers and non-smokers. *Psychopharmacol.* **146,** 455-464 (1999).

Nater, U. M., Krebs, M. & Ehlert, U. Sensation seeking, music preference, and psychophysiological. reactivity to music. *Musicae Scientiae* **9,** 239-254 (2005).

O'Jile, J. R., Ryan, L. M., Parks-Levy, J., Betz, B. & Gouvier, W. D. Sensation seeking and risk behaviors in young adults with and without a history of head injury. *Applied Neuropsychol.* **11,** 107-112 (2004).

Ongen, D. E. (2007). The relationships between sensation seeking and gender role orientations among Turkish university students. *Sex Roles*, *57*(1-2), 111-118. doi: 10.1007/s11199-007-9214-4

Perez, J. & Torrubia, R. Sensation seeking and antisocial behavior in a student sample. *Pers. Indiv. Diff. 6*, 401-403 (1985).

Perez, J. & Torrubia, R. Feasibility and validity of the Spanish version of the Sensation-Seeking Scale (Form V). *Revista Latinoamericana De Psicologia* **18,** 7-& (1986).

Perkins, K. A. *et al.* Initial nicotine sensitivity in humans as a function of impulsivity. *Psychopharmacol.* **200,** 529-544 (2008).

Pfefferbaum, B. & Wood, P. B. Self-report study of impulsive and delinquent behavior in college students. *J. Adolesc. Health* **15,** 295-302 (1994).

Rahmani, S. & Lavasani, M. G. Gender Differences in Five Factor Model of Personality and Sensation Seeking. *Procedia-Social and Behavioral Sciences* **46,** 2906-2911(2012).

Rawlings, D. Personality correlates of liking for 'unpleasant' paintings and photographs. *Pers. Indiv. Diff.* **34,** 395-410 (2003).

Ridgeway, D. & Russell, J. A. Reliability and validity of the Sensation Seeking Scale: Psychometric problems in Form V. *J. Consult. Clin. Psychol.* **48,** 662-664 (1980).

Ripa, C. P. L., Hansen, H. S., Mortensen, E. L., Sanders, S. A. & Reinisch, J. M. A Danish version of the Sensation Seeking Scale and its relation to a broad spectrum of behavioral and psychological characteristics. *Pers. Indiv. Diff.* **30,** 1371-1386 (2001).

Roberti, J. W. Personality characteristics of undergraduates with career interests in forensic identification. *Journal of Employment Counseling,* **41,** 117-125 (2004).

Rosenblitt, J. C., Soler, H., Johnson, S. E. & Quadagno, D. M. Sensation seeking and hormones in men and women: Exploring the link. *Horm. Behav.* **40,** 396-402 (2001).

Rowland, G., Fouts, G. & Heatherton, T. Television viewing and sensation seeking : Uses, preferences and attitudes. *Pers. Indiv. Diff.* **10,** 1003-1006 (1989).

Rowland, G. L. & Franken, R. E. The 4 dimensions of sensation seeking: A confirmatory factor analysis. *Pers. Indiv. Diff. 7*, 237-240 (1986).

Rowland, G. L., Franken, R. E., Williams, S. E. & Heatherton, T. The perception of sensation seeking in familiar and unfamiliar others. *Pers. Indiv. Diff. 9*, 237-241 (1988).

Santelli, J., Bernstein, D. M., Zborowski, L. & Bernstein, J. M. Pursuing and distancing, and related traits: A cross-cultural assessment. *J. Pers. Assess.* **55,** 663-672 (1990).

Sasaki, H. & Kanachi, M. The effects of trial repetition and individual characteristics on decision making under uncertainty. *J. Psychol.* **139,** 233-246 (2005).

Satinder, K. P. & Black, A. Cannabis use and sensation-seeking orientation. *J. Psychol.* **116,** 101-105 (1984).

Schroth, M. L. Effects of extrinsic conditions on measurement of sensation seeking. *Percep. Motor Skills* **70,** 315-320 (1990).

Schroth, M. L. Dyadic adjustment and sensation seeking compatibility. *Pers. Indiv. Diff.* **12,** 467-471 (1991).

Schroth, M. L. A comparison of sensation seeking among different groups of athletes and nonathletes. *Pers. Indiv. Diff.* **18,** 219-222 (1995).

Steenkamp, J. & Baumgartner, H. The role of optimum stimulation level in exploratory consumer behavior. *J. Consumer Res.* **19,** 434-448 (1992).

Terasaki, M. & Imada, S. Sensation seeking and food preferences. *Pers. Indiv. Diff.* **9,** 87-93 (1988).

Thomas, D. A. Measuring volunteers for exciting psychology experiments with the Sensation Seeking Scale. *J. Personality Assess.* **53,** 790-801 (1989).

Thornquist, M. H., Zuckerman, M. & Exline, R. V. Loving, liking, looking and sensation seeking in unmarried college couples. *Pers. Indiv. Diff.* **12,** 1283-1292 (1991).

Tonetti, L. *et al.* Morningness-eveningness preference and sensation seeking. *Europ. Psychiat.* **25,** 111-115 (2010).

Torrubia, R., Avila, C., Molto, J. & Caseras, X. The Sensitivity to Punishment and Sensitivity to Reward Questionnaire (SPSRQ) as a measure of Gray's anxiety and impulsivity dimensions. *Pers. Indiv. Diff.* **31**, 837-862 (2001).

van Wijk, C. H. Sensation-seeking personality traits of navy divers. *Diving and Hyperbaric Medicine* **37,** 10-15 (2007).

Vodanovich, S. J. & Kass, S. J. Age and gender differences in boredom proneness. *J. Soc. Behav. Personal.* **5,** 297-307 (1990).

Voracek, M., Tran, U. S. & Dressler, S. G. Digit ratio (2D:4D) and sensation seeking: New data and meta-analysis. *Pers. Indiv. Diff. 48,* 72-77 (2010).

Vuust, P. *et al.* Personality influences career choice: sensation seeking in professional musicians. *Music Educ. Res.* **12,** 219-230 (2010).

Watt, J. D. & Ewing, J. E. Toward the development and validation of a measure of sexual boredom. *J. Sex Res.* **33,** 57-66 (1996).

White, H. R. & Johnson, V. Risk taking as a predictor of adolescent sexual activity and use of contraception. *J. Adol. Res.* **3,** 317-331 (1988).

Zacny, J. P. A possible link between sensation-seeking status and positive subjective effects of oxycodone in healthy volunteers. *Pharmacol. Biochem. Behav.* **95,** 113-120 (2010).

Zuckerman, M., Eysenck, S. & Eysenck, H. J. Sensation seeking in England and America: Cross-cultural, age, and sex comparisons. *J. Consult. Clin. Psychol.* **46,** 139-149 (1978).

Zuckerman, M., Kuhlman, D. M., Thornquist, M. & Kiers, H. 5 (or 3) robust questionnaire scale factors of personality without culture. *Pers. Indiv. Diff. 12*, 929-941 (1991).

Zuckerman, M. & Litle, P. Personality and curiosity about morbid and sexual events. *Pers. Indiv. Diff.* **7,** 49-56 (1986).

Zuckerman, M. & Neeb, M. Demographic influences in sensation seeking and expressions of sensation seeking in religion, smoking and driving habits. *Pers. Indiv. Diff.* **1,** 197-206 (1980).

3 – Men's and women's scores by study year

Total SSS-V scores have declined very slightly over time (Figure S1). This trend reaches significance in the men's scores (B = -0.071, SE = 0.025, $p < .01$, Beta = -0.358, R^2 = 0.128) and is only marginal for the women's (B = -0.055, SE = 0.031, p = .08, Beta = -0.239, R^2 = 0.057); however, only a very small amount of the variance in the data is accounted for by publication date, and the regression slopes do not differ significantly from each other (z = 0.4, p = .69). TAS scores are presented in the main text. For Dis, both men's scores (B = -0.026, SE = 0.012, $p < .05$, Beta = -0.273, R^2 = 0.075) and women's scores (B = -0.025, SE = 0.013, p = .056, Beta = -0.255, R^2 = 0.065) scores have also declined slightly over time. Male and female scores for the other sub-scales have remained stable over time (BS: men, B = -0.009, SE = 0.009, n.s.; women, B = -0.008, SE = 0.009, n.s.; ES: men: B = -0.00004, SE = 0.012, n.s.; women: B = 0.006, SE = 0.012, n.s.).

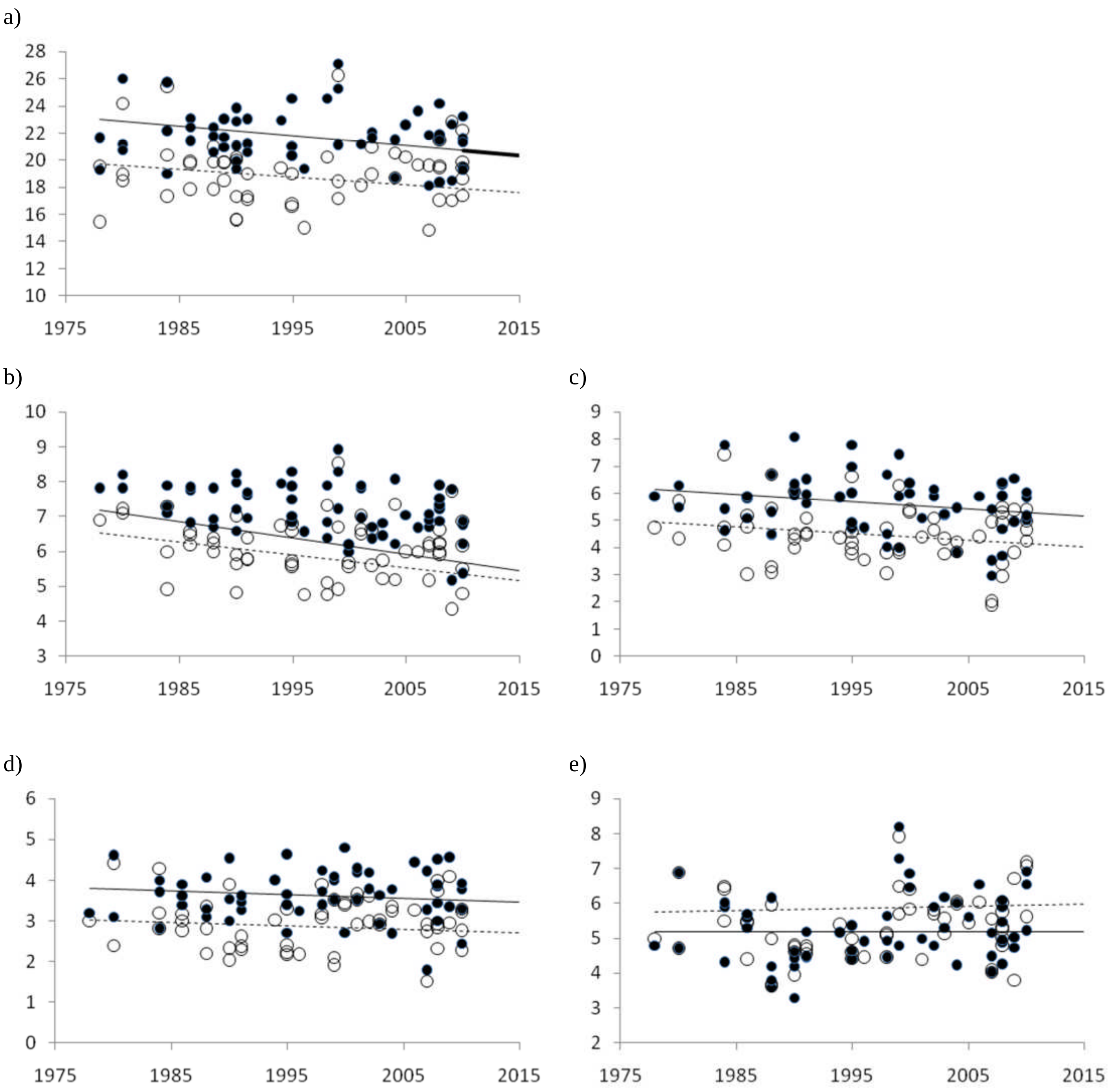

Figure S1 Men's (filled circles, solid lines) and women's (open circles, dashed lines) sensation-seeking scores by study year: a) Total; b) TAS; c) Dis; d) BS; e) ES.

4 – Funnel plots and tests for publication bias for all subscales

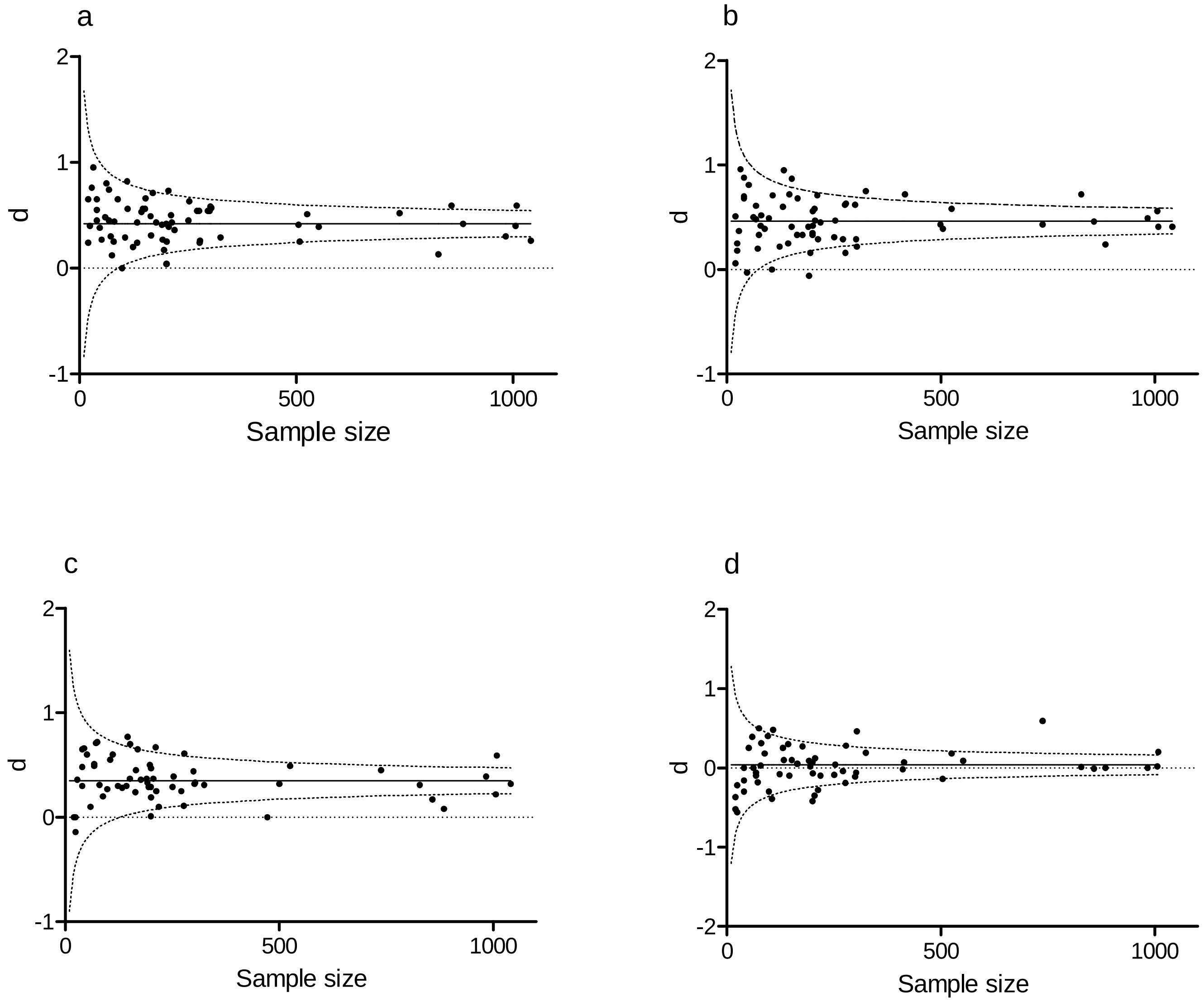

Figure S2 Effect sizes by sample size. a) TAS; b) Dis; c) BS; d) ES (for Total, see main text)

Tests for publication bias.

Effect size was not correlated with inverse variance for any of the subscales (TAS: Kendall's tau-b = -0.15, n.s.; Dis: tau-b = -0.00, n.s. ; BS: tau-b = -0.07, n.s.; ES: tau-b = 0.11, n.s.).

5 – Variance Ratios

We examined whether the variance in sensation-seeking scores differs between the sexes, as higher male than female variability has been reported for other aspects of personality (Archer & Mehdikhani, 2003; Borkenau et al., 2013).

We obtained the variance ratio (VR) where available for each study, by dividing the variance in male scores by the variance in female scores (a VR of 1 therefore indicates equal variances, greater than one indicates greater variance in male scores and less than one indicates greater variance in female scores). We then log-transformed the variance ratios for each study and used a one-sample t-test with a test value of zero on the transformed VRs to determine whether the VR was significantly different from one.

The variance ratios showed no distinct pattern: variance was greater in women than in men for the SSS-V Total (0.92; $t_{(57)} = 2.70$, $p = .012$), and the TAS (0.83; $t_{(55)} = 5.12$, $p = <.001$), Dis showed no sex difference in variance (1.04, n.s.), BS showed significantly greater male variance (1.15; $t_{(53)}= 2.92$, $p = .005$), and ES showed no sex difference (1.07, n.s.). Variance ratios >1 indicate greater male variance, and variance ratios <1 greater female variance.

References

Archer, J. & Mehdikhani, M. Variability among males in sexually selected attributes. *Rev. Gen. Psychol.* ***7,*** 219–236 (2003).

Borkenau, P., Martina, H., Kuppens, P., Realo, A. & Allik, J. Sex differences in variability in personality: a study in four samples. *J. Personal.* **81,** 49-60 (2013).
